# “After reducing alcohol, things now work well at home”: Perceived impacts of the *Mlambe* intervention on alcohol use, relationship dynamics, household economics, and HIV treatment adherence in Malawian couples

**DOI:** 10.1371/journal.pone.0331202

**Published:** 2025-08-29

**Authors:** Sarah A. Gutin, Nancy Mulauzi, Jane Jere, Cobbener Sungani, Scott Tebbetts, Allison Ruark, James Mkandawire, Amy A. Conroy

**Affiliations:** 1 Department of Community Health Systems, School of Nursing, University of California San Francisco, San Francisco, California, United States of America; 2 Division of Prevention Sciences, School of Medicine, University of California San Francisco, San Francisco, California, United States of America; 3 Invest in Knowledge, Zomba, Malawi; 4 University of California Berkeley, Berkeley, California, United States of America; 5 Biological and Applied Health Sciences, Wheaton College, Wheaton, Illinois, United States of America; Makere University School of Public Health, UGANDA

## Abstract

Unhealthy alcohol use is prevalent in sub-Saharan Africa and can worsen poverty, couple relationships, and HIV treatment outcomes. In response, we assessed participant experiences with *Mlambe*, a pilot study of an economic and relationship-strengthening intervention for couples living with unhealthy alcohol use and HIV. Exit interviews were conducted with a subset of 20 couples who participated in a pilot trial of *Mlambe* in Zomba, Malawi. A 10-month intervention consisted of financial literacy and couples counseling sessions and incentivized savings accounts. Eligible couples were married, ≥ 18, with at least one partner with unhealthy alcohol use (according to AUDIT-C screen), and currently on antiretroviral therapy (ART). Interviews were recorded, transcribed, translated, and analyzed using dyadic framework analysis. Intervention couples reported that alcohol use caused many problems and that reducing or quitting use brought improvements to their physical and emotional health, and the well-being of their marriages and households. Before *Mlambe*, couples reported that quarrels about alcohol use were common*.* After the intervention, couples reported improvements to their relationships and less alcohol use, which led to more open and respectful couple communication and other marital improvements including increased sexual satisfaction and trust. Women and men described that post-intervention, men reduced spending on alcohol which improved availability of money for household needs such as food and clothing, and that feeling more economically secure reduced stress and led to a more “peaceful family.” After *Mlambe*, male drinkers described more motivation to pursue income-generating activities, and that reduced alcohol use led to greater medication adherence as they no longer forgot to take ART when drinking. *Mlambe* may contribute to positive change for couple relationships and health behaviors through mechanisms including reduced conflict and poverty related to reduced alcohol use. The model appears promising for couples in resource-poor settings where HIV, poverty, and alcohol use are mutually reinforcing.

## Introduction

Unhealthy alcohol use is highly prevalent in sub-Saharan Africa and can worsen economic conditions, couple relationships, and health [1]. Unhealthy alcohol use is defined as consuming alcohol above limits that are safe for health (this is defined by the US National Institute on Alcohol Abuse and Alcoholism as drinking no more than 2 drinks a day for men, and no more than one drink for women) (NIAAA). Economic insecurity, unemployment, and low-wage, stressful jobs are linked to unhealthy alcohol use [2–5]. Alcohol use can also indirectly affect health by damaging couple relationships that are needed for social support and overall wellbeing [6–10]. Research with couples has found that partners can influence each other’s alcohol use, and alcohol use can cause and result in relationship distress including intimate partner violence (IPV) [11–13].

Unhealthy alcohol use is a health threat for people living with HIV (PLWH) [14–16] and unhealthy drinking is higher among PLWH as compared to the general population [17,18]. Among PLWH, high levels of alcohol use are associated with lower antiretroviral therapy (ART) adherence and poor clinical HIV care outcomes [15,19–25]. Drinking behaviors are often gendered, with unhealthy alcohol use being more common among men compared to women [25–27]. In Malawi, men are the primary alcohol users; among men who drink (30% of men), 51% meet the criteria for unhealthy drinking [28]. Unhealthy alcohol use by one partner can also negatively influence the health of partners who do not drink. In a study from Malawi, men had difficulty taking HIV medications when they were drinking, and their female partners (most of whom did not drink) also faced adherence challenges when their husband’s drinking led to IPV and food insecurity [10].

Whereas the negative impacts of alcohol use on relationships are myriad, couple relationships also offer an opportunity to leverage social support within the couple to mitigate the harms of unhealthy drinking [29]. Studies that have used a relationship-strengthening framework have been successful in sub-Saharan Africa at reducing alcohol use and addressing HIV-related behaviors [30–34]. In South Africa, interventions focused on strengthening relationships among heterosexual couples have led to reductions in alcohol use and increased uptake of HIV testing [30,31]. However, most alcohol interventions have intervened at the individual level and have not adequately considered the interplay of social and behavioral factors for committed couples, despite research that suggests an urgent need for interventions that consider alcohol use as a couple-level issue involving and affecting both partners [29,35,36].

Alcohol use can also worsen conditions of poverty. Malawi’s poverty levels are high with 71% of adults living on $2 USD or less per day [37]. Only 34% of Malawians have access to the formal banking sector for which financial literacy is a major barrier [38]. In response to the syndemics of of poverty, HIV, IPV, and alcohol use [13,39,40], we developed, implemented, and then evaluated *Mlambe*, an economic and relationship-strengthening intervention. *Mlambe* consisted of incentivized savings accounts with financial literacy strengthening activities and couples counseling sessions for couples with unhealthy alcohol use and living with HIV in Zomba, Malawi [41,42].

**Fig 1:**
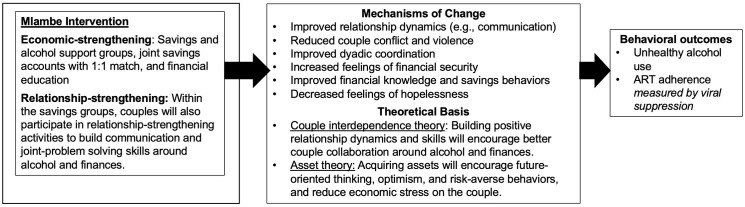
Conceptual model for intervention effects on behavior change.

*Mlambe* was guided by a conceptual model based on the couple interdependence theory of communal coping [43] and asset theory [44,45]. Couple interdependence theory stipulates that improved relationship dynamics, including couple communication, reduced couple conflict and violence, increased partner support around reducing alcohol use, and financial literacy and planning would improve couple functioning and in turn lead to reductions in unhealthy alcohol use (Fig 1). Asset theory suggests that having assets (e.g., a savings account, education, or a small business) can change household economic status but also attitudes, behaviors, and hope for the future [44,45]. Having assets can also create an “asset effect” which leads to expectations for more assets, creating a sense of security and safety, and encouraging more optimism and planning for the future.

Following the *Mlambe* trial, we conducted exit interviews to triangulate findings, better evaluate and understand the main trial results, and confirm hypotheses around the key areas of change. Interviews explored domains drawn from the couple interdependence theory of communal coping [43], asset theory [44,45], and syndemic theory [39,40]. Briefly, in the quantitative intervention component, we found that *Mlambe* participants reported drinking less alcohol and had lower rates of unhealthy alcohol use, improved ART adherence, and increased viral suppression [42]. In addition, couples in the intervention compared to control reported greater increases in couple communication, sexual satisfaction, intimacy, unity, and trust over time and reported decreases in emotional and physical IPV [46]. The qualitative exit interviews were critical to understanding the trends in intervention effects but also to understanding the unexpected ways that the intervention impacted couple relationships and health outcomes [47,48]. Using our intervention conceptual model (Fig 1), the present qualitative study aimed to describe couples’ experiences with the *Mlambe* intervention and perceptions of how *Mlambe* may have changed relationship dynamics, family finances, alcohol use, and adherence to ART.

## Methods

The study took place in the Zomba district of Malawi where 15% of adults are living with HIV [49]. Participants were recruited between May 24, 2021 and November 16, 2021 at an urban, public HIV clinic at the district hospital; a rural, private community hospital; and a peri-urban, public health center.

For the larger trial, couples were eligible if: 1) in a non-polygamous marital union; (2) aged 18 or older; (3) had at least one partner with a positive AUDIT-C screen (score of ≥4 for men and ≥3 for women; prior 3 months) and on ART for at least six months (referred to as the “index patient” to distinguish between dyad members). If either dyad member was living with HIV, they must have disclosed their HIV status to the spouse. We excluded couples who reported severe IPV in the past 3 months based on the WHO domestic violence module [50] and/or feared that their safety would be at risk through participation.

Study staff presented information on the study at health information talks in the waiting rooms of the HIV clinics while patients were attending appointments and picking up medications. Index patients could approach the recruiters if interested. Patients who arrived after the health talks were approached directly by recruiters. If the patient was interested and eligible, recruiters gave patients an information card to give to their spouse and set up a phone appointment to screen the partner. If the partner was also interested and eligible, the couple was scheduled for their first appointment to obtain written, informed consent in the local language of Chichewa.

From the larger trial sample of 78 couples (equally divided between study arms), we selected 20 couples (15 from the intervention arm and five from the control arm) to take part in exit interviews. Couples were purposively sampled to obtain an equal balance across the recruitment sites. We had aimed to select couples based on low or high satisfaction and participation levels in the trial, however, we had 100% participation and 100% satisfaction in the trial. The results presented focus on the responses from the 15 intervention couples who took part in exit interviews.

### Intervention

*Mlambe* has been described elsewhere [41,42] but in brief, *Mlambe* consisted of ten monthly intervention sessions delivered by a pair of facilitators. The facilitators, one male and one female, had backgrounds in education and counseling and delivered the intervention sessions as a team. Prior to the first session, couples set up a joint banking account at a national bank in which they could start making deposits. Couples were incentivized with a 1:1 match for each Malawi Kwacha saved, up to $10 USD per month. Sessions took place in community-based locations equipped with a conference room or at a rented space at HIV clinics. A structured manual was followed and was divided into sessions on economic and relationship-strengthening activities, which included sessions on banking services, budgeting, savings and asset building, debt management, relationship and power dynamics, and couple communication skills. All sessions were group-based except for two one-on-one couples counselling sessions to learn and practice relationship skills. In the last session, community-based extension workers employed by the Malawian government were invited to meet the couples and advise them on best practices for their business goals (e.g., animal husbandry, growing vegetables for a produce stand) and continued to meet with couples as needed at their homes to offer support. To start their family-based income-generating activity (IGA), couples could access the combined savings and matched component and invest the money into the business of their choice.

### Data collection

Trained, gender-matched interviewers with prior qualitative skills and experience discussing sensitive topics conducted the exit interviews in the local language of Chichewa. To improve comfort discussing sensitive topics, participants were interviewed by research staff that they were familiar with and with whom they had developed rapport. The research staff had already provided the intervention sessions and multiple rounds of surveys to the interview participants. Both partners were interviewed separately but simultaneously in private rooms to ensure confidentiality, to minimize bias, and to ensure that participants felt free to express their personal view independent from their partner. Interviews were conducted following the completion of all intervention sessions at 10 months post-intervention. Based on our *a priori* conceptual model of how *Mlambe* improves health and relationships, we followed a semi-structured interview guide that asked open-ended questions framed around the theories that the intervention was based on (Asset Theory and Interdependence Theory) [43–45]. Interview questions focused on overall experiences and perceptions (both positive and negative) of *Mlambe*, knowledge and skills gained, perceived impacts of *Mlambe* on relationship dynamics (including closeness, sexual life, arguments), household economics and food insecurity, adherence to medications and HIV care, mental health and well-being, and suggestions for how *Mlambe* could be improved. Similar questions were asked of both partners and interviews lasted approximately 60–90 minutes.

### Analysis

The interviews were audio-recorded, transcribed, and translated from Chichewa to English by research assistants who are fluent in both languages. The research manager performed spot-checks to ensure that translations were through and accurate. We conducted an individual-level analysis and dyadic-level analysis using a framework analysis approach, which allows for comparative analysis of themes and cases by utilizing data matrices [51]. We read and reviewed the interview transcript pairs, created summary tables, and organized the data by couples and by the larger categories from the interview guide (e.g., perceived impacts on alcohol use). We then coded for patterns within each larger category by comparing responses within and between couples by examining raw quotes and noting any agreements or disagreements between partners (e.g., the husband reported that he quit consuming alcohol, but the wife said he only reduced alcohol use). A team of five coders, which included representation from Malawi and are included as authors, coded the full set of transcripts and met regularly to build consensus around definitions and applications of codes and to discuss the findings using the summary tables and the themes that emerged. Inter-coder reliability was not formally assessed but discrepancies in code application among the research team were discussed to reach consensus. Finally, we held community meetings with key stakeholders including couples who participated to present the findings and ensure our interpretations were correct.

### Ethics statement

This study received ethical approval from the Human Research Protection Program at the University of California San Francisco and the National Health Science Research Committee in Malawi. The study was registered at ClinicalTrials.gov (NCT #04906616). Written informed consent was collected twice, once for enrollment into the trial and again for participation in the exit interviews.

## Results

### Sample characteristics

Among the 40 individuals who completed an exit interview, mean age was 45 years and most (80%) had primary education or less. At baseline, 62.5% reported severe food insecurity. Couples had been together for a mean of 14 years and reports of IPV in the past 12 months were common, with 30% reporting emotional, 20% reporting physical, and 17.5% reporting sexual IPV. Most couples (74%) were sero-concordant positive and among all individuals on ART, 61% reported 95–100% adherence to treatment (Table 1).

**Table 1:** Baseline characteristics of the *Mlambe* exit interview sample (N = 15 couples; 30 individuals).

Variable	Overall
	%, Mean (SD)
Age (years; range: 27–72)	45.4 (12.5)
Primary school education or less	83.0
Severe food insecurity	68.5
Relationship duration (years)	12.9 (10.1)
Sero-concordant positive	60.0
Physical IPV (any)	10.0
Sexual IPV (any)	13.3
Emotional IPV (any)	27.0
**Main index patient with alcohol use (N = 15)**	
AUDIT-C score (range: 3–12)	3.8 (4.3)
Number of drinking days (range: 0–30)	3.6 (6.7)
Self-reported 95–100% adherence to ART	80.0
**All partners on ART**	
Time on ART (years)	8.6 (3.6)

IPV=Intimate partner violence; SD=Standard Deviation

We highlight five themes that emerged regarding perceived changes of *Mlambe* on: 1) alcohol use patterns, 2) improvements in couple relationships, 3) improvements in sexual life and sexual satisfaction, 4) improvements in economic security, and 5) improvements in ART adherence.

Intervention couples reported that alcohol use caused many problems in the past, and that reducing or quitting alcohol use because of *Mlambe* brought about multiple improvements to their physical and emotional health, and the well-being of their marriages and households. While couples were asked about any negative feedback and how *Mlambe* could be improved in the future, the findings highlight largely positive perceptions of the intervention.

### Changes in alcohol use patterns and underlying motivations for drinking

Many men and women believed that because of *Mlambe*, men’s alcohol use decreased or changed in other positive ways. While some men reported stopping drinking completely, others said they reduced their intake or switched to drinking alcoholic beverages with lower alcohol content like *chibuku* [a commercially manufactured sorghum beer with low alcohol content] instead of *kachasu* [a locally distilled spirit made from maize with a high alcohol content]. Some men also described changes to their drinking patterns, from consuming alcohol at formal drinking establishments (i.e., village bars) to drinking at home. Some wives viewed this as a positive change because they could monitor what and how much their partners drank.

*I said, “my partner you should reduce your alcohol use” … Even if he was told [to reduce] he was not reducing his alcohol use but since Mlambe started he has improved, he has reduced his alcohol use.* -Woman, 34 years old

*After Mlambe I am now a changed man, by … 6:00 pm I’m already home, my partner even asks, “Are you back this time?” I would say yes, sometimes I take alcohol while at home, something I never used to do. -*Man, 44 years old

Many women and men felt that since men had reduced alcohol use, they were now coming home earlier and contributing to family life. Participants reported that before *Mlambe,* men would often go out to drinking establishments and some would be so drunk that they would sleep at the bar and not come home. However, both women and men noted that because of *Mlambe*, some men spent fewer nights out drinking and sleeping outside the home.

*My partner used to drink alcohol a lot and sometimes he was even sleeping at the bottle store, but now he stopped doing that since we started this program.* -Woman, 41 years old

This increased presence of men at home often meant that men were now contributing to a family business or taking part in household chores, while also creating space for couples to spend more time together and communicate about family life.

*He is not moving out as he used to, he has reduced. He was leaving for beer drinking places in the morning and was coming back in the evening, he was going back in the morning. So, I have noticed that he has reduced this, he can go to his friends to chat and come back in time, he is able to do household chores without any problem. -*Woman, 34 years old

While most men and women reported a reduction in men’s alcohol use because of *Mlambe*, there were some discrepancies among couples about whether the men actually reduced their use. One woman reported that her husband had stopped drinking alcohol while he only reported reducing his alcohol intake. In addition, three men reported that they either reduced or stopped drinking alcohol while their wives reported that they had only reduced their consumption or had not altered their behavior at all, as seen in the quotes below from a husband and wife.

*Sometimes I would take 4–5 drinks … so they asked me what I benefit when I take alcohol, so now I have reduced to a smaller number of drinks, maybe 3–4 … so that time I was a drunkard [local term referring to a person who is publicly intoxicated frequently], after Mlambe I am now a changed man.* -Man, 44 years old

*Imagine telling him to quit alcohol and then he replies, “ah I can’t stop alcohol, it’s better we should divorce”. You see that, so it’s difficult, because when you are telling one to stop something, it means it’s bad. Everything learned, he was leaving it behind at the exit door of the sessions.* -Woman, 33 years old

Participants described several key sources of motivation that helped them to change alcohol use patterns in positive ways, including social support. First, men reported receiving consistent advice and reminders from their wives to reduce their alcohol use. Many men reported that these reminders, together with communication skills taught during *Mlambe* sessions, helped them to reduce or stop drinking alcohol. Second, men described feeling motivated to reduce their alcohol consumption because of support they received from other peers in the intervention, who advised them to reduce excessive drinking. Participants recalled how one of the lessons of *Mlambe* was to save money on alcohol to develop a small business. As a result, many men said they developed a desire to save their money or invest it in a business. Other men explained that the savings aspect of *Mlambe* and the business or income-generating activities they started kept them busy and as a result, they reported drinking less. Some men described how *Mlambe* helped them realize that spending money on alcohol was wasteful and caused them to use sparse resources on alcohol instead of on needed household items or food.

*They enlightened me that you lose a lot of money with alcohol intake, such that when you want to make a budget you just be wasting.* -Man, 40 years old

### Perceived improvements to couple relationships

Most couples reported that their relationships improved because of *Mlambe*. Couples explained that some improvements in relationship quality were due to participation in the intervention and related to learning communication skills or learning to approach their partner with respect. Other improvements in the relationship were seen as being prompted by men’s reduction in alcohol use.

Men and women reported that quarrels and family conflicts about alcohol use were common prior to *Mlambe.* Couples also reported that, before *Mlambe*, talking about alcohol use within their relationship was rare. Many of the quarrels stemmed from men spending all their money or household funds on alcohol that could have been used for food or school fees for children. However, after the intervention, couples reported that they had learned about reducing alcohol use for the benefit of the entire family. Couples were usually in agreement on positive changes to their marriages and both men and women reported quarreling less because of *Mlambe*.

*He went to drink alcohol and spent all the money. He came back home without any money. This is when we started arguing. We were arguing frequently. When someone did not speak well … we ended up quarrelling. But this stopped after we joined this project. -Woman, 34 years old*

*What happens is that you use that money for beer and [you] finish the money. So when you get home you find that there are arguments … sometimes I would speak roughly [to my wife]. But … we should be respecting each other as you have taught us. So I see that these days we are able to understand each other more. – Man, 70 years old*

Men and women reported that communication skills that they learned as part of *Mlambe* helped to facilitate more open and respectful couple communication and other marital improvements, including more active listening and reports of increased trust. Couples felt that these skills helped to strengthen relationships and they felt more “free with each other”. Where in the past, discussing alcohol use or family finances had been off limits, couples reported that they could now openly discuss these topics, and they felt heard by each other. Couples discussed learning to approach each other with humility and respect, and to use “I statements” rather than casting blame with “you statements”. Men and women said these techniques helped to reduce fighting in the relationship and led more than one couple to say that *Mlambe* had brought peace to their family. As one husband and wife explained, the communication skills they learned helped to improve communication in their relationship.

*We have benefited from this in our family, we understand each other and when speaking to each other, when I am angry with something, I start talking to her, “I am upset when…” And my partner listens and understands me … so because of this, since we started those sessions in our family, we have peace every time.* -Man, 49 years old

*On communicating with my spouse, we learnt that if I want to talk to him, I should not blame him like using ‘you,’ no I should say ‘I’ because if I say I … that the person who is listening is also interested that she has said ‘I’ and is not blaming me … the other thing is that when we are communicating, we must understand each other and be humble to each other. When one is speaking it is good that one listens to what the partner is speaking rather than both of us speaking at once, you end up quarrelling. – Woman, 39 years old*

However, in some couples, it appeared that men were presenting a more positive view of improvements to communication since reports by their wives did not coincide with what they said.

### Perceived improvements in sexual life and sexual satisfaction

While sexual satisfaction was not a primary focus of the *Mlambe* intervention, we anticipated that there could be spillover effects on sexual satisfaction through improvements in other relationship domains (e.g., communication, trust) and through reductions in alcohol use. While a few participants admitted that nothing changed with their sexual life, or that they already had a healthy sexual life to begin with, most intervention couples reported improvements in either the quality or quantity of sex. Improvements to sexual satisfaction were believed to be related to several shifts in the relationship that occurred through participation in *Mlambe*.

Both men and women reported that before *Mlambe* when men were drinking, they often had extra-marital sexual partners and that these two behaviors went hand-in-hand. Women reported how their husbands were now spending less time “sleeping outside the house” (meaning having other sexual partners) and at drinking establishments, and more time at home. Some husbands admitted that they were having many sexual partners (referred to by the men as “womanizing”) because of their unhealthy drinking. After *Mlambe*, men reported less drinking, fewer sexual partners, and being more faithful to their wives. After taking part in *Mlambe*, one husband explained, “*I had a secret lover but I ended the relationship after I noticed that I was not doing good to my partner because every time she was having stress.”* Some women specifically explained that as their husbands decreased their drinking and were more likely to be sober, having sex was more enjoyable. Prior to *Mlambe*, women would either refuse to have sex when their husbands were drunk or sex would not happen because he was too tired or could not perform sexually. Husbands also acknowledged this was happening. For example, one husband admitted that:

*When I have gone to drink alcohol, I might fail to do sex so she might be sad since I will be tired. So you just come back and lay on the bed, nothing happens as if you are sharing a bed with a fellow man.* -Man, 40 years old

Couples also described how *Mlambe* taught them to communicate more openly and respectfully with their partner, which extended into the sexual realm. Communication around sex covered topics such as values around monogamy (e.g., addressing desires or temptations for other sexual partners), sexual desires and needs (e.g., how often sex should happen), and acceptable reasons for refusing sex (e.g., when sick). A husband described how he and his wife learned about the importance of verbal communication and learning to read each other’s signs for wanting sex:

*Mlambe has helped us in this because in the past when we wanted to have sex, it was just happening as a surprise [laughing] and Mlambe has told us that when you want to have sex with your wife you need to show signs so that she is also aware what her husband wants.* -Man, 60 years old

Finally, sexual life improved when sex was perceived as consensual and equally desired by both partners. This contrasted with the past, when one partner would pressure or force the other to have sex or refuse sex, often because the man was drinking. Two men explained that before *Mlambe*, they were forcing their wives to have sex with them, but since taking part in the intervention, they had stopped that behavior.

### Perceived improvements in economic security

Both partners described how men would use household finances to buy alcohol before *Mlambe*, which left them unable to support basic family needs. Women and men both described situations where men spent money on alcohol that was being saved for buying food or for paying children’s school fees. Some men and women reported that their children were going hungry while the men used what money they had to buy alcohol. After *Mlambe*, men reduced spending on alcohol which led to more money being available to meet basic needs, such as buying more food, soap, clothing, fertilizer, or paying school fees for children. Couples were also able to save to plant vegetable gardens or make house improvements, such as buying iron sheets that could replace old roofs. When men were not using family funds to buy alcohol, this led to fewer conflicts, more “peace in the household”, a greater sense of economic security and reduced financial stress and led couples to have a more positive view of the future, with one participant saying that saving money helped to “transform” her household.

*When he left to drink alcohol, he left home without giving me money to buy food, and he came back while he was drunk and then he asked for food. I told him that there was no food and then he blamed me for not preparing food yet he did not give me any money and he spent his money on alcohol.* -Woman, 39 years old

*Before Mlambe, I wasn’t able to save, I wasn’t able to take care of my family, but after I joined Mlambe I started saving because they taught us that, I am able to support my family, before when my wife tells me that flour is done, I was saying I cannot use this money, is meant for alcohol, but now that doesn’t happen.* –Man, 44 years old

After *Mlambe*, women and men described how they were able to invest in small businesses such as rearing livestock (goats, pigs, and chickens), growing vegetables for sale, opening a small salon, or buying a motorbike that could be used as a local taxi. Prior to *Mlambe*, some men described being “idle” but after *Mlambe*, male drinkers described being more motivated to pursue income-generating opportunities such as rearing livestock.

*He was saying that he was drinking alcohol because he doesn’t have things to do, when he found things to do [business] he was not going to drink alcohol because he was busy with the business.* -Woman, 30 years old

*Through the business we are able to find money and its different from just being idle then anything can come into mind like “I should go to the beer hall”. -*Man, 69 years old

However, in some couples, even though men said they had reduced their alcohol use, women reported that they were “saving alone” and starting small businesses on their own without their partner’s support.

Couples also described that prior to *Mlambe*, men oversaw finances in the household and women had little say when men spent their savings on drinking. As one woman described, before *Mlambe*, her husband, “took all the money and ruled over it”. Another man described that when his wife asked him for money, he would scoff at her and say, *“I am the one who earns money, and you do not have any right to tell me what to do”*. However, *Mlambe* included a session on balancing power dynamics including economic power, and after *Mlambe*, women reported decisions about how to spend household finances became more equitable. Men often brought home their earnings and either developed a budget with their wives or discussed how to spend or save the money with their wives.

*The other skill I learnt is on finances, because previously, every one of us was keeping their money alone and that also meant spending, but now, we are able to discuss expenditure.* –Man, control arm, 46 years old

### Perceived improvements in adherence to ART

Men and women both described how prior to *Mlambe*, men experienced ART adherence challenges because of alcohol use. For many men, they forgot to take medications altogether or took them late because of drinking. Women confirmed that alcohol intake was affecting the medication adherence of their partners. Couples also noted that at times it was hard to get to facilities for appointments and to collect medications because they were busy. As a result of *Mlambe* sessions, however, men and their wives reported that they started to reduce their alcohol use, and this led to greater medication adherence and men feeling healthier. Couples reported that they learned in counselling sessions that medication adherence can help them live longer, improve their immunity, and their overall health. Many men identified that skipping their medications was a direct result of drinking too much. As one man explained, *“[I] no longer skip taking medication because I stopped drinking alcohol. The skipping of medication was as a result of alcohol intake.”*

*Before Mlambe, he was drinking heavily, he was spending carelessly. I would advise him but he wouldn’t listen … but he reduced all that after Mlambe, and also right now he is complying with medication prescriptions … we were arguing because he was declining taking medication but now, he is taking medication well. -*Woman, 35 years old

In addition to providing general support for medication adherence, an important way that couples supported each other was through reminders to take their ART on time and to not miss doses. Both men and women reported that they now remind their partners to attend clinic appointments and many reported that they collect medications for each other if one person is sick or busy. Many couples also talked about creating daily reminders whereby their partners reminded them to take their medications every morning before leaving for work, or right after they eat breakfast. Women also reminded their partners that taking medications and alcohol at the same time was not good for their health. The *Mlambe* sessions helped couples to see each other as “guardians” or each other’s health. Many participants also felt that improved communication skills led them to support each other to take their ART after *Mlambe*.

*When I wake up in the morning, immediately after taking let’s say porridge, she comes and give me the medicine, then I take the medicine with water. When I forget she reminds me, so I don’t skip taking medicine.* -Man, 44 years old

*If someone forgets to take the ARVs the other one should remind him. So we help each other, asking each other if he has taken the ARVs. If you have not yet taken them you give thanks for the reminder and then go and take them.* -Woman, 72 years old

## Discussion

These findings highlight the multiple mechanisms by which *Mlambe* may contribute to positive change for couple relationships and health behaviors related to HIV and alcohol use. Reductions in alcohol use may lead to improvements in couple relationship quality, family finances, and engagement with HIV treatment and care. In *Mlambe*, we holistically examined family health by using a qualitative approach to better understand how reductions in alcohol use affect the daily lives of couples and families. We found a complicated picture of the interwoven pathways and mechanisms by which a reduction in alcohol use improved multiple outcomes including relationship quality and health. The *Mlambe* model appears promising in resource-poor settings with couples affected by HIV where poverty may be a driver of alcohol use.

Couples were asked to make suggestions about how the intervention could be improved to better meet their needs, and asked to give positive and negative feedback, but mostly reported positive perceptions of the intervention. This aligns with our quantitative trial data finding high levels of feasibility and acceptability of *Mlambe* [42]. Similar to *Mlambe* qualitative data, quantitative data also found reductions in alcohol use, high ART adherence as measured through viral load testing, improvements in relationship quality, and greater equitable financial decision-making within couples [42,46,52]. A strength of this qualitative approach is that these positive results triangulate and largely confirm the quantitative results but also add depth and nuance to our understanding of how *Mlambe* led to positive change for couples.

Research with couples has shown that partners can influence each other’s alcohol use [11,53], yet few studies in sub-Saharan Africa have used a relationship-strengthening framework to reduce alcohol use [30]. *Mlambe* is one of the few couple-level interventions that target alcohol use alongside economic issues and has shown reductions in unhealthy alcohol use [42]. Most men qualitatively reported either reducing or stopping alcohol use altogether and the positive effects that this had on other areas including relationship quality and family finances. Similar to our *Mlambe* findings, other studies in sub-Saharan Africa that have used a relationship-strengthening framework have been successful at reducing alcohol use and addressing HIV-related behaviors. For example, in South Africa, interventions focused on strengthening couple relationships among heterosexual couples have led to reductions in alcohol use and increased uptake of HIV testing [30,31]. Studies have also found that relationship-strengthening interventions can reduce IPV [54,55]. In Rwanda, a gender-transformative couple-level intervention found that improvements in relationship dynamics such as couple communication and emotional closeness as well as reducing alcohol use emerged as an important mechanism for reducing IPV [56]. In South Africa, a couples intervention focused on reducing unhealthy alcohol use also found decreases in IPV [57]. Therefore, *Mlambe* findings add to a growing literature in sub-Saharan Africa that show that a relationship-strengthening framework has great promise for reducing alcohol use in this context.

Participants also described how *Mlambe* had enhanced couple relationships through improved communication and more equitable and shared decision-making in the home. *Mlambe* improved relationship quality due to fewer family conflicts, more open and respectful communication between partners generally and in particular about alcohol use and helped develop active listening skills and trust. Prior research from sub-Saharan African countries has also found that improvements in relationship dynamics, such as communication, commitment, trust, and unity can improve various health outcomes including HIV testing and status disclosure, ART adherence, uptake and retention in HIV care [5,31,33,34,58–61] and reductions in experienced and perpetrated IPV and reduced sexual coercion [62]. HIV behavioral interventions have also been developed for couples and have focused on enhancing couple relationship dynamics for improved family health outcomes [31,58,63,64]. While less research has focused on alcohol use, a study among couples in South Africa found that unhealthy alcohol use was positively associated with poor relationship dynamics such as negative communication styles and mistrust [65]. Decision-making power, or feeling that relationships are more equitable, may also help to improve relationship dynamics, which in turn can improve couple support for reductions in alcohol use. A study with couples in South Africa supports this and found that shared power was strongly associated with higher relationship quality [66]. The effect of power dynamics on relationship quality remains a crucial study area due to its potential impact on couples’ alcohol-related behaviors [43,66–68].

*Mlambe* couples reported improvements in sexual satisfaction that resulted from reductions in alcohol use. This in turn led to more enjoyable, frequent, consensual, and sober sex, and increased open communication around sexual needs and desires. Sexual satisfaction is an important dimension of relationship quality [69] and data from a study conducted in Malawi and Eswatini found that couples with higher sexual satisfaction also report higher relationship quality and lower negative relationship experiences like reports of emotional, physical, or sexual IPV [70]. This is also in line with our finding that couples reporting improvements in sexual satisfaction because of *Mlambe* also experienced less coercive sex and more open sexual communication. While few studies have examined sexual satisfaction in the context of alcohol use, a study from Uganda found lower sexual satisfaction when men consumed alcohol [71,72]. While these findings are in the opposite direction, they reinforce our *Mlambe* finding that sexual satisfaction is positively associated with reductions in alcohol use. Sexual satisfaction has also been associated with positive outcomes such as lower anticipated HIV stigma [73] and may be a promising dimension to work on in couple-level interventions as a way to build stronger, more supportive and resilient relationships.

The *Mlambe* intervention focused on strengthening relationships while also offering incentivized savings accounts and financial literacy training. Expectedly, *Mlambe* couples reported that their family finances improved, but the financial training and savings accounts also led to less alcohol consumption. Finances that previously would have been spent on alcohol were described as being spent on household needs. While economic strengthening interventions in sub-Saharan Africa have been found to reduce sexual risk-taking among adolescents and to improve suppression of HIV among adolescents living with HIV [74–76] using such interventions to reduce unhealthy alcohol use, as *Mlambe* did, is novel. *Mlambe* couples also reported more equitable financial decision-making in their families. This is a novel finding, as most economic strengthening and micro-savings interventions have focused just on women rather than including couples as a unit [41,77–79]. By offering financial literacy training to both members of the couple together, it seems that *Mlambe* led to a sense of joint financial decision-making. It may also be that by working with couples, men were less likely to view women’s involvement in financial matters as a threat to their role as providers and as the heads of households [41,80,81]. Couples reported how a reduction in alcohol use also led to more money being available for starting small family businesses. While some couples appeared to be developing small businesses together, in others, women were running the business alone. While this still injects needed resources into the family, traditional gender roles around the division of labor in the household may have been in play, with running a business becoming yet another task that women had to be responsible for.

The association between alcohol use and ART non-adherence is well established in SSA [82–89]. In line with this literature, *Mlambe* couples described improvements in ART adherence because of reductions in alcohol use. Specifically, *Mlambe* couples supported each other through reminders about taking treatment and not missing doses. This is in line with data from other African settings that also supports the positive impact that partner support can have on treatment adherence. In the area of prevention of mother to child transmission (PMTCT) of HIV, a lack of partner support has been noted as a reason why women stop ART [90,91] while partner support has been associated with ART adherence among PMTCT users in Ethiopia and Tanzania and viewed as a necessary precondition for ART initiation and adherence [92,93]. While partners can interfere with treatment adherence, positive relationship attributes such as unity, satisfaction, intimacy, commitment, and partner support have been found to be associated with reported adherence [35,94]. Primary partners can provide critical social support to people living with HIV and can be leveraged, as we saw in *Mlambe*, to provide ART adherence support in a population with challenges around alcohol use.

While both men and women were generally in agreement that *Mlambe* had positive effects and helped to reduce alcohol use, improve couple communication, sexual satisfaction, family finances, and treatment adherence, there were some disagreements among couples about the magnitude of impact. Overall, men tended to report more change or improvement than women. For example, while men might report abstaining from alcohol, their wives would report that men had reduced their alcohol intake but had not quit completely. Another area of disagreement was around financial saving. Sometimes men reported that they were now saving money with their wives, but their wives reported that they were saving without help from their husbands. Therefore, while the wives were still reporting improvements, they were often less than those reported by men. These differences between men and women in reporting about the level of positive effects experienced because of *Mlambe* are similar to a study from Malawi which found that men and women often had conflicting accounts of their family financial situation and the level and impacts of men’s alcohol use. However, when reporting on sexual satisfaction, it was more common for women to report improvements in this area, while men reported that things were the same as before. This qualitative finding is similar to our quantitative results which found larger positive effects of *Mlambe* on sexual satisfaction among women compared to men [95]. It is possible that for women, reductions in alcohol use by their husbands led to a more fulfilling sexual life but that men did not perceive these changes in the same way. Overall, while there was some variation, it seems that both men and women benefitted from *Mlambe* and perceived the intervention as creating positive change in their lives.

### Limitations

This study has some limitations. As with all research, it is possible that participants were overly positive in their interviews about intervention satisfaction given their relationship with the *Mlambe* study, their desire to maintain a good relationship with the study, and the hope to access future opportunities. However, we had checks in place to overcome this potential bias. During the exit interviews, the interviewers highlighted the importance of hearing both positive and negative feedback and focused on the importance of improving the intervention in the future. However, the positive qualitative findings are consistent with the quantitative results which also showed very positive experiences with *Mlambe* [96]. Therefore, while we had intended to include results in these analyses from those reporting low satisfaction and those reporting negative experiences, everyone reported being satisfied or very satisfied with the intervention. Social desirability bias is also a concern. Participants may have under-reported or misreported details about alcohol use or changes in alcohol use, behavior change related to extra-marital sexual partnerships, changes in relationship quality, or changes in their financial behavior. In this case, having the accounts of both partners is critical as sometimes one partner reported changes while the other reported some or no change. The inclusion of the wives’ voices in the findings also helps to offset overly positive portrayals of behavior change among the men—who were the primary target of the alcohol intervention.

## Conclusions

These qualitative findings highlight multiple mechanisms by which *Mlambe* may contribute to positive change for couple relationships and health behaviors related to HIV and alcohol use. The *Mlambe* model appears promising in resource-poor settings with couples affected by HIV and should be scaled up to improve relationship dynamics, family finances, and adherence to ART while reducing alcohol use. While the *Mlambe* intervention was designed for heterosexual couples living with HIV, it is possible to adapt the intervention for various populations. If the intervention was being offered to couples not affected by HIV, the HIV content could be removed. Also, *Mlambe* participants were middle-aged on average and were in established, married couples. The intervention could also be adapted for younger couples, but they might have other relationship dynamics that should be targeted. However, many relationship dynamics, such as building trust and couple communication, are likely to benefit any couple. Improving the family unit is a strong foundation on which to improve health outcomes and as seen in *Mlambe*, can lead to positive downstream effects not just for the person who drinks alcohol – but for the whole family.

## References

[1] World Health Organization. Global status report on alcohol and health 2018. World Health Organization; 2019.

[2] Kalichman SC, Simbayi LC, Kagee A, Toefy Y, Jooste S, Cain D, et al. Associations of poverty, substance use, and HIV transmission risk behaviors in three South African communities. Soc Sci Med. 2006;62(7):1641–9. doi: 10.1016/j.socscimed.2005.08.021 16213078

[3] Dunkle KL, Jewkes RK, Brown HC, Gray GE, McIntryre JA, Harlow SD. Transactional sex among women in Soweto, South Africa: prevalence, risk factors and association with HIV infection. Soc Sci Med. 2004;59(8):1581–92. doi: 10.1016/j.socscimed.2004.02.003 15279917

[4] Setlalentoa B, Pisa P, Thekisho G, Ryke E, Loots Du T. The social aspects of alcohol misuse/abuse in South Africa. South Afr J Clin Nutr. 2010;23(sup2):11–5.

[5] Conroy AA, Ruark A, McKenna SA, Tan JY, Darbes LA, Hahn JA, et al. The unaddressed needs of alcohol-using couples on antiretroviral therapy in Malawi: formative research on multilevel interventions. AIDS Behav. 2020;24(6):1599–611. 31456201 10.1007/s10461-019-02653-y PMC7044068

[6] Shamu S, Abrahams N, Temmerman M, Musekiwa A, Zarowsky C. A systematic review of African studies on intimate partner violence against pregnant women: prevalence and risk factors. PLoS One. 2011;6(3):e17591. doi: 10.1371/journal.pone.0017591 21408120 PMC3050907

[7] Woolf-King SE, Maisto SA. Alcohol use and high-risk sexual behavior in Sub-Saharan Africa: a narrative review. Arch Sex Behav. 2011;40(1):17–42. 19705274 10.1007/s10508-009-9516-4

[8] Homish GG, Leonard KE, Kearns-Bodkin JN. Alcohol use, alcohol problems, and depressive symptomatology among newly married couples. Drug Alcohol Depend. 2006;83(3):185–92. doi: 10.1016/j.drugalcdep.2005.10.017 16337752 PMC1783684

[9] Kerr RB. Food security in northern Malawi: gender, kinship relations and entitlements in historical context. J Southern African Stud. 2005;31(1):53–74.

[10] Conroy AA, McKenna SA, Ruark A. Couple interdependence impacts alcohol use and adherence to antiretroviral therapy in Malawi. AIDS Behav. 2019;23(1):201–10. doi: 10.1007/s10461-018-2275-2 30218319 PMC7232967

[11] Rodriguez LM, Neighbors C, Knee CR. Problematic alcohol use and marital distress: an interdependence theory perspective. Addict Res Theory. 2014;22(4):294–312.

[12] Bello B, Moultrie H, Somji A, Chersich MF, Watts C, Delany-Moretlwe S. Alcohol use and sexual risk behaviour among men and women in inner-city Johannesburg, South Africa. BMC Public Health. 2017;17(Suppl 3):548. doi: 10.1186/s12889-017-4350-4 28832283 PMC5498865

[13] Hatcher AM, Gibbs A, McBride RS, Rebombo D, Khumalo M, Christofides NJ. Gendered syndemic of intimate partner violence, alcohol misuse, and HIV risk among peri-urban, heterosexual men in South Africa. Soc Sci Med. 2022;295:112637. 31708236 10.1016/j.socscimed.2019.112637 PMC7296316

[14] Myers B, Lombard C, Joska JA, Abdullah F, Naledi T, Lund C, et al. Associations between patterns of alcohol use and viral load suppression amongst women living with HIV in South Africa. AIDS Behav. 2021;25(11):3758–69. 33876383 10.1007/s10461-021-03263-3 PMC8560660

[15] Velloza J, Kemp CG, Aunon FM, Ramaiya MK, Creegan E, Simoni JM. Alcohol use and antiretroviral therapy non-adherence among adults living with HIV/AIDS in Sub-Saharan Africa: a systematic review and meta-analysis. AIDS Behav. 2020;24(6):1727–42. doi: 10.1007/s10461-019-02716-0 31673913 PMC7190427

[16] Ferreira-Borges C, Rehm J, Dias S, Babor T, Parry CDH. The impact of alcohol consumption on African people in 2012: an analysis of burden of disease. Trop Med Int Health. 2016;21(1):52–60. doi: 10.1111/tmi.12618 26448195

[17] Duko B, Ayalew M, Ayano G. The prevalence of alcohol use disorders among people living with HIV/AIDS: a systematic review and meta-analysis. Subst Abuse Treat Prev Policy. 2019;14(1):52. doi: 10.1186/s13011-019-0240-3 31727086 PMC6854786

[18] Park LS, Hernández-Ramírez RU, Silverberg MJ, Crothers K, Dubrow R. Prevalence of non-HIV cancer risk factors in persons living with HIV/AIDS: a meta-analysis. AIDS. 2016;30(2):273–91. doi: 10.1097/QAD.0000000000000922 26691548 PMC4689318

[19] Hahn JA, Samet JH. Alcohol and HIV disease progression: weighing the evidence. Curr HIV/AIDS Rep. 2010;7(4):226–33. 20814765 10.1007/s11904-010-0060-6 PMC2938419

[20] Heestermans T, Browne JL, Aitken SC, Vervoort SC, Klipstein-Grobusch K. Determinants of adherence to antiretroviral therapy among HIV-positive adults in sub-Saharan Africa: a systematic review. BMJ Glob Health. 2016;1(4):e000125. doi: 10.1136/bmjgh-2016-000125 28588979 PMC5321378

[21] Scott-Sheldon LA, Carey KB, Johnson BT, Carey MP, Team MR. Behavioral interventions targeting alcohol use among people living with HIV/AIDS: a systematic review and meta-analysis. AIDS Behav. 2017;21(2):126–43. 28831609 10.1007/s10461-017-1886-3 PMC5660648

[22] Williams EC, Hahn JA, Saitz R, Bryant K, Lira MC, Samet JH. Alcohol use and human immunodeficiency virus (HIV) infection: current knowledge, implications, and future directions. Alcohol Clin Exp Res. 2016;40(10):2056–72. doi: 10.1111/acer.13204 27696523 PMC5119641

[23] Braithwaite RS, Bryant KJ. Influence of alcohol consumption on adherence to and toxicity of antiretroviral therapy and survival. Alcohol Res Health. 2010;33(3):280–7. 23584069 PMC3860503

[24] Salmon-Ceron D, Lewden C, Morlat P, Bévilacqua S, Jougla E, Bonnet F, et al. Liver disease as a major cause of death among HIV infected patients: role of hepatitis C and B viruses and alcohol. J Hepatol. 2005;42(6):799–805. doi: 10.1016/j.jhep.2005.01.022 15973779

[25] Miller AP, Pitpitan EV, Kiene SM, Raj A, Jain S, Zúñiga ML, et al. Alcohol use and alcohol-related consequences are associated with not being virally suppressed among persons living with HIV in the Rakai region of Uganda. Drug Alcohol Depend. 2021;228:109005. doi: 10.1016/j.drugalcdep.2021.109005 34600249 PMC8628865

[26] He Z, Bishwajit G, Yaya S. Prevalence of alcohol and tobacco use among men and women in Namibia. Int J Environ Res Public Health. 2018;16(1):59. doi: 10.3390/ijerph16010059 30587825 PMC6338963

[27] Chang LW, Grabowski MK, Ssekubugu R, Nalugoda F, Kigozi G, Nantume B, et al. Heterogeneity of the HIV epidemic in agrarian, trading, and fishing communities in Rakai, Uganda: an observational epidemiological study. Lancet HIV. 2016;3(8):e388–96. doi: 10.1016/S2352-3018(16)30034-0 27470029 PMC4973864

[28] World Health Organization. Global status report on alcohol and health. Geneva, Switzerland: World Health Organization; 2014.

[29] Conroy AA, McKenna SA, Leddy A, Johnson MO, Ngubane T, Darbes LA, et al. “If she is drunk, i don’t want her to take it”: partner beliefs and influence on use of alcohol and antiretroviral therapy in South African couples. AIDS Behav. 2017;21(7):1885–91. doi: 10.1007/s10461-017-1697-6 28150121 PMC5493498

[30] Wechsberg WM, Zule WA, El-Bassel N, Doherty IA, Minnis AM, Novak SD, et al. The male factor: outcomes from a cluster randomized field experiment with a couples-based HIV prevention intervention in a South African township. Drug Alcohol Depend. 2016;161:307–15. doi: 10.1016/j.drugalcdep.2016.02.017 26946991 PMC5645020

[31] Darbes LA, McGrath NM, Hosegood V, Johnson MO, Fritz K, Ngubane T, et al. Results of a couples-based randomized controlled trial aimed to increase testing for HIV. J Acquir Immune Defic Syndr. 2019;80(4):404–13. 30730356 10.1097/QAI.0000000000001948 PMC6524952

[32] Turan J, Kwena Z, Owuor K, Hatcher AM, Helova A, Braun T, et al. Efficacy of home visits for pregnant couples to promote couple HIV testing and family health. San Francisco: CROI; 2025.

[33] Hampanda K, Helova A, Odwar T, Odeny T, Onono M, Bukusi E, et al. Male partner involvement and successful completion of the prevention of mother-to-child transmission continuum of care in Kenya. Int J Gynaecol Obstet. 2021;152(3):409–15. doi: 10.1002/ijgo.13442 33108671 PMC7902296

[34] Rogers AJ, Achiro L, Bukusi EA, Hatcher AM, Kwena Z, Musoke PL, et al. Couple interdependence impacts HIV-related health behaviours among pregnant couples in southwestern Kenya: a qualitative analysis. J Int AIDS Soc. 2016;19(1):21224. doi: 10.7448/IAS.19.1.21224 27887669 PMC5124108

[35] Conroy A, Leddy A, Johnson M, Ngubane T, van Rooyen H, Darbes L. “I told her this is your life”: relationship dynamics, partner support and adherence to antiretroviral therapy among South African couples. Cult Health Sex. 2017;19(11):1239–53. doi: 10.1080/13691058.2017.1309460 28398134 PMC5626574

[36] Conroy AA, Mckenna SA, Ruark A. Couple interdependence impacts alcohol use and adherence to antiretroviral therapy in Malawi. AIDS Behav. 2018. 10.1007/s10461-018-2275-2 PMC7232967 30218319

[37] World Bank Group. Republic of Malawi Poverty Assessment. 2017. https://openknowledge.worldbank.org/handle/10986/26488

[38] UNCTAD. Access to Financial Services in Malawi: Policies and Challenges. 2014.

[39] Singer M. A dose of drugs, a touch of violence, a case of AIDS, part 2: further conceptualizing the SAVA syndemic. Free Inq Creat Sociol. 2006;34(1):39–53.

[40] Singer M. A dose of drugs, a touch of violence, a case of AIDS: conceptualizing the SAVA syndemic. Free Inq Creat Sociol. 1996;24(2):99–110.

[41] Conroy AA, Tebbetts S, Darbes LA, Hahn JA, Neilands TB, McKenna SA, et al. Development of an economic and relationship-strengthening intervention for alcohol drinkers living with HIV in Malawi. AIDS Behav. 2023;27(7):2255–70. doi: 10.1007/s10461-022-03956-3 36520335 PMC9753077

[42] Conroy AA, Hahn JA, Neilands TB, Darbes LA, Tebbetts S, Mulauzi N. Pilot trial results of Mlambe: an economic and relationship-strengthening intervention to address heavy drinking and adherence to antiretroviral therapy in Malawi. AIDS Behav. 2024. 10.1007/s10461-024-04326-x PMC11587313 38551718

[43] Lewis MA, McBride CM, Pollak KI, Puleo E, Butterfield RM, Emmons KM. Understanding health behavior change among couples: an interdependence and communal coping approach. Soc Sci Med. 2006;62(6):1369–80. doi: 10.1016/j.socscimed.2005.08.006 16146666

[44] Schreiner M, Sherraden MW. Can the poor save?: Saving & asset building in individual development accounts. New Brunswick, N.J.: Transaction. 2007.

[45] Sherraden M. Stakeholding: Notes on a theory of welfare based on assets. Soc Serv Rev. 1990;64(4):580–601.

[46] Ruark A, Tebbetts S, Darbes L, Hahn JA, Neilands TB, Mulauzi N. Mlambe economic and relationship-strengthening intervention increases relationship quality and decreases violence among couples in Malawi. In: AIDS Impact, Stockholm, Sweden, 2023.

[47] Lewis S, Romano C, De Bruecker G, Murrough JW, Shelton R, Singh JB, et al. Analysis of clinical trial exit interview data in patients with treatment-resistant depression. Patient. 2019;12(5):527–37. doi: 10.1007/s40271-019-00369-8 31270774

[48] Gnanasakthy A, DeMuro C, Clark M, Mordin M, Thomas S. Role of patient-reported outcome measures in the assessment of central nervous system agents. Ther Innov Regul Sci. 2013;47(5):613–8. 30235577 10.1177/2168479013495686

[49] MDHS. Malawi demographic and health survey 2015-16. Maryland: NSO and ORC Macro; 2016.

[50] World Health Organization. Researching violence against women: practical guidelines for researchers and activists. World Health Organization; 2005.

[51] Ritchie J, Lewis J, Nicholls CM, Ormston R. Qualitative research practice: a guide for social science students and researchers. Sage. 2013.

[52] Gopalakrishnan L, Mulauzi N, Mkandawire J, Ssewamala FM, Tebbetts S, Neilands TB, et al. Effects of economic empowerment and relationship strengthening intervention on financial behaviors among couples living with HIV: The Mlambe pilot trial in Malawi. SSM Popul Health. 2025;29:101768. doi: 10.1016/j.ssmph.2025.101768 40104040 PMC11919304

[53] Levitt A, Cooper ML. Daily alcohol use and romantic relationship functioning: evidence of bidirectional, gender-, and context-specific effects. Pers Soc Psychol Bull. 2010;36(12):1706–22. doi: 10.1177/0146167210388420 21098471

[54] Starmann E, Collumbien M, Kyegombe N, Devries K, Michau L, Musuya T, et al. Exploring couples’ processes of change in the context of SASA!, a violence against women and HIV prevention intervention in Uganda. Prev Sci. 2017;18(2):233–44. doi: 10.1007/s11121-016-0716-6 27682273 PMC5243896

[55] Stern E, Willan S, Gibbs A, Myrttinen H, Washington L, Sikweyiya Y, et al. Pathways of change: qualitative evaluations of intimate partner violence prevention programmes in Ghana, Rwanda, South Africa and Tajikistan. Cult Health Sex. 2021;23(12):1700–16. doi: 10.1080/13691058.2020.1801843 32896204

[56] Doyle K, Levtov RG, Karamage E, Rakshit D, Kazimbaya S, Sayinzoga F, et al. Long-term impacts of the Bandebereho programme on violence against women and children, maternal health-seeking, and couple relations in Rwanda: a six-year follow-up of a randomised controlled trial. EClinicalMedicine. 2023;64:102233. doi: 10.1016/j.eclinm.2023.102233 37781160 PMC10539919

[57] Minnis AM, Doherty IA, Kline TL, Zule WA, Myers B, Carney T, et al. Relationship power, communication, and violence among couples: results of a cluster-randomized HIV prevention study in a South African township. Int J Womens Health. 2015;7:517–25. doi: 10.2147/IJWH.S77398 25999767 PMC4435250

[58] Hatcher AM, Darbes L, Kwena Z, Musoke PL, Rogers AJ, Owino G, et al. Pathways for HIV prevention behaviors following a home-based couples intervention for pregnant women and male partners in Kenya. AIDS Behav. 2020;24(7):2091–100. doi: 10.1007/s10461-019-02774-4 31894444 PMC7319865

[59] Hampanda K, Abuogi L, Musoke P, Onono M, Helova A, Bukusi E, et al. Development of a novel scale to measure male partner involvement in the prevention of mother-to-child transmission of HIV in Kenya. AIDS Behav. 2020;24(1):291–303. doi: 10.1007/s10461-019-02546-0 31152357 PMC6885105

[60] Conroy AA. “It means there is doubt in the house”: perceptions and experiences of HIV testing in rural Malawi. Cult Health Sex. 2014;16(4):397–411. doi: 10.1080/13691058.2014.883645 24580127 PMC3991518

[61] Conroy AA. The influence of relationship power dynamics on HIV testing in rural Malawi. J Sex Res. 2015;52(3):347–59. doi: 10.1080/00224499.2014.883590 24670263 PMC4177026

[62] Dunkle K, Stern E, Chatterji S, Heise L. Effective prevention of intimate partner violence through couples training: a randomised controlled trial of Indashyikirwa in Rwanda. BMJ Glob Health. 2020;5(12):e002439. doi: 10.1136/bmjgh-2020-002439 33355268 PMC7757483

[63] Turan JM, Darbes LA, Musoke PL, Kwena Z, Rogers AJ, Hatcher AM, et al. Development and piloting of a home-based couples intervention during pregnancy and postpartum in Southwestern Kenya. AIDS Patient Care STDS. 2018;32(3):92–103. doi: 10.1089/apc.2017.0285 29620927 PMC5865625

[64] Wechsberg WM, El-Bassel N, Carney T, Browne FA, Myers B, Zule WA. Adapting an evidence-based HIV behavioral intervention for South African couples. Subst Abuse Treat Prev Policy. 2015;10:6. doi: 10.1186/s13011-015-0005-6 25888856 PMC4344778

[65] Woolf-King SE, Conroy AA, Fritz K, Johnson MO, Hosegood V, van Rooyen H. Alcohol use and relationship quality among South African couples. Subst Use Misuse. 2018;:1–10. 10.1080/10826084.2018.1531428 PMC6487654 30407888

[66] Conroy AA, McGrath N, van Rooyen H, Hosegood V, Johnson MO, Fritz K, et al. Power and the association with relationship quality in South African couples: Implications for HIV/AIDS interventions. Soc Sci Med. 2016;153:1–11. doi: 10.1016/j.socscimed.2016.01.035 26859436 PMC4788545

[67] Crankshaw TL, Voce A, Butler LM, Darbes L. Expanding the relationship context for couple-based HIV prevention: Elucidating women’s perspectives on non-traditional sexual partnerships. Soc Sci Med. 2016;166:169–76. doi: 10.1016/j.socscimed.2016.08.020 27566046 PMC5023493

[68] Robles TF, Slatcher RB, Trombello JM, McGinn MM. Marital quality and health: a meta-analytic review. Psychol Bull. 2014;140(1):140–87. doi: 10.1037/a0031859 23527470 PMC3872512

[69] Stanik CE, Bryant CM. Sexual satisfaction, perceived availability of alternative partners, and marital quality in newlywed African American couples. J Sex Res. 2012;49(4):400–7. doi: 10.1080/00224499.2011.568127 21516593 PMC3289721

[70] Conroy AA, Ruark A, Neilands TB, Darbes LA, Johnson MO, Tan JY, et al. Development and validation of the couple sexual satisfaction scale for HIV and sexual health research. Arch Sex Behav. 2021;50(7):3297–311. doi: 10.1007/s10508-021-02098-2 34609644

[71] Kajubi P, Ruark A, Hearst N, Ruteikara S, Green EC. Assessment of an HIV-prevention intervention for couples in peri-urban Uganda: pervasive challenges to relationship quality also challenge intervention effectiveness. Afr J AIDS Res. 2020;19(3):249–62. doi: 10.2989/16085906.2020.1811357 33119459 PMC8336899

[72] Ruark A, Kajubi P, Ruteikara S, Green EC, Hearst N. Couple relationship functioning as a source or mitigator of HIV risk: associations between relationship quality and sexual risk behavior in Peri-urban Uganda. AIDS Behav. 2018;22(4):1273–87. doi: 10.1007/s10461-017-1937-9 29090396 PMC5878978

[73] Gutin SA, Ruark A, Darbes LA, Neilands TB, Mkandawire J, Conroy AA. Supportive couple relationships buffer against the harms of HIV stigma on HIV treatment adherence. BMC Public Health. 2023;23(1):1878. doi: 10.1186/s12889-023-16762-w 37770885 PMC10540419

[74] Ssewamala FM, Han C-K, Neilands TB. Asset ownership and health and mental health functioning among AIDS-orphaned adolescents: findings from a randomized clinical trial in rural Uganda. Soc Sci Med. 2009;69(2):191–8. doi: 10.1016/j.socscimed.2009.05.019 19520472 PMC2819297

[75] Bermudez LG, Ssewamala FM, Neilands TB, Lu L, Jennings L, Nakigozi G, et al. Does economic strengthening improve viral suppression among adolescents living with HIV? Results from a cluster randomized trial in Uganda. AIDS Behav. 2018;22(11):3763–72. 29846836 10.1007/s10461-018-2173-7 PMC6204092

[76] Ksoll C, Lilleør HB, Lønborg JH, Rasmussen OD. Impact of village savings and loan associations: evidence from a cluster randomized trial. J Dev Econ. 2016;120:70–85. doi: 10.1016/j.jdeveco.2015.12.003

[77] Dworkin SL, Blankenship K. Microfinance and HIV/AIDS prevention: assessing its promise and limitations. AIDS Behav. 2009;13(3):462–9. 19294500 10.1007/s10461-009-9532-3 PMC3770268

[78] Leatherman S, Dunford C, Metcalfe M, Reinsch M, Gash M. Integrating Microfinance and Health: Benefits, challenges and reflections for moving forward. Global Microcredit Summit; 2011 November 14–17, 2011.

[79] MacPherson E, Sadalaki J, Nyongopa V, Nkhwazi L, Phiri M, Chimphonda A. Exploring the complexity of microfinance and HIV in fishing communities on the shores of Lake Malawi. Rev Afr Polit Econ. 2015;42(145):414–36.

[80] Dunbar MS, Maternowska MC, Kang M-SJ, Laver SM, Mudekunye-Mahaka I, Padian NS. Findings from SHAZ!: a feasibility study of a microcredit and life-skills HIV prevention intervention to reduce risk among adolescent female orphans in Zimbabwe. J Prev Interv Community. 2010;38(2):147–61. doi: 10.1080/10852351003640849 20391061 PMC4578719

[81] Slegh H, Barker G, Kimonyo A, Ndolimana P, Bannerman M. I can do women’s work: reflections on engaging men as allies in women’s economic empowerment in Rwanda. Gend Dev. 2013;21(1):15–30.

[82] Azar MM, Springer SA, Meyer JP, Altice FL. A systematic review of the impact of alcohol use disorders on HIV treatment outcomes, adherence to antiretroviral therapy and health care utilization. Drug Alcohol Depend. 2010;112(3):178–93. doi: 10.1016/j.drugalcdep.2010.06.014 20705402 PMC2997193

[83] Kalichman S, Banas E, Kalichman M, Mathews C. Stigmatisation of alcohol use among people receiving antiretroviral therapy for HIV infection, Cape Town, South Africa. Glob Public Health. 2020;15(7):1040–9. doi: 10.1080/17441692.2020.1724314 32053472

[84] Lancaster KE, Lungu T, Mmodzi P, Hosseinipour MC, Chadwick K, Powers KA, et al. The association between substance use and sub-optimal HIV treatment engagement among HIV-infected female sex workers in Lilongwe, Malawi. AIDS Care. 2017;29(2):197–203. doi: 10.1080/09540121.2016.1211244 27442009 PMC5138102

[85] Macleod D, Shanaube K, Skalland T, Limbada M, Mandla N, Bwalya J, et al. Viral suppression and self-reported ART adherence after 3 years of universal testing and treatment in the HPTN 071 (PopART) community-randomised trial in Zambia and South Africa: a cross-sectional analysis. Lancet HIV. 2022;9(11):e751–9. doi: 10.1016/S2352-3018(22)00237-5 36332652 PMC9646982

[86] Shubber Z, Mills EJ, Nachega JB, Vreeman R, Freitas M, Bock P, et al. Patient-reported barriers to adherence to antiretroviral therapy: a systematic review and meta-analysis. PLoS Med. 2016;13(11):e1002183. doi: 10.1371/journal.pmed.1002183 27898679 PMC5127502

[87] Ayieko P, Kisanga E, Mshana G, Nkosi S, Hansen CH, Parry CDH, et al. Epidemiology of alcohol use and alcohol use disorders among people living with HIV on antiretroviral therapy in Northwest Tanzania: implications for ART adherence and case management. AIDS Care. 2024;36(5):652–60. doi: 10.1080/09540121.2023.2299324 38295268

[88] Parcesepe AM, Nash D, Tymejczyk O, Reidy W, Kulkarni SG, Elul B. Gender differences and psychosocial factors associated with problem drinking among adults enrolling in HIV care in Tanzania. AIDS Behav. 2019;23(6):1612–22. doi: 10.1007/s10461-018-2340-x 30465107 PMC6529300

[89] Chang GC, West CA, Kim E, Low AJ, Lancaster KE, Behel SS. Hazardous alcohol use and HIV indicators in six African countries: results from the population-based HIV impact assessments, 2015-2017. J Int AIDS Soc. 2022;25(11):e26029. 10.1002/jia2.26029 PMC9677379 36408717

[90] Kim MH, Zhou A, Mazenga A, Ahmed S, Markham C, Zomba G, et al. Why did i stop? Barriers and facilitators to uptake and adherence to ART in Option B+ HIV care in Lilongwe, Malawi. PLoS One. 2016;11(2):e0149527. doi: 10.1371/journal.pone.0149527 26901563 PMC4762691

[91] Chadambuka A, Katirayi L, Muchedzi A, Tumbare E, Musarandega R, Mahomva AI, et al. Acceptability of lifelong treatment among HIV-positive pregnant and breastfeeding women (Option B+) in selected health facilities in Zimbabwe: a qualitative study. BMC Public Health. 2017;18(1):57. doi: 10.1186/s12889-017-4611-2 28743251 PMC5526299

[92] Geremew H, Geremew D, Abdisa S, Dessie AM, Kassa GM, Moges NA. Adherence to option B PMTCT program and its predictors among HIV-positive women in Ethiopia. Health Sci Rep. 2023;6(7):e1404. 10.1002/hsr2.1404 PMC10323164 37425229

[93] Zacharius KM, Basinda N, Marwa K, Mtui EH, Kalolo A, Kapesa A. Low adherence to Option B antiretroviral therapy among pregnant women and lactating mothers in eastern Tanzania. PLoS One. 2019;14(2):e0212587. 10.1371/journal.pone.0212587 PMC6386496 30794633

[94] Conroy AA, McKenna S, Ruark A, Neilands TB, Spinelli M, Gandhi M. Relationship dynamics are associated with self-reported adherence but not an objective adherence measure in Malawi. AIDS Behavior. 2022;26(11):3551–62. 35507094 10.1007/s10461-022-03636-2 PMC9631807

[95] Conroy AA, Ruark A, Mulauzi N, Mkandawire J, Darbes LA, Hahn JA, et al. Mlambe economic and relationship-strengthening intervention for alcohol use decreases violence and improves relationship quality in couples living with HIV in Malawi. Soc Sci Med. 2024;362:117407. doi: 10.1016/j.socscimed.2024.117407 39405663 PMC11585424

[96] Conroy AA, Hahn JA, Neilands TB, Darbes LA, Tebbetts S, Mulauzi N, et al. Pilot trial results of mlambe: an economic and relationship-strengthening intervention to address heavy drinking and adherence to antiretroviral therapy in Malawi. AIDS Behav. 2024;28(7):2296–306. doi: 10.1007/s10461-024-04326-x 38551718 PMC11587313

